# Analysis of Long Period Gratings Inscribed by CO_2_ Laser Irradiation and Estimation of the Refractive Index Modulation

**DOI:** 10.3390/s20226409

**Published:** 2020-11-10

**Authors:** Ana M. Rocha, Ana I. Machado, Telmo Almeida, Joana Vieira, Margarida Facão

**Affiliations:** 1 Instituto de Telecomunicações and University of Aveiro, Campus Universitário de Santiago, 3810-193 Aveiro, Portugal; anaimachado@ua.pt (A.I.M.); telmopelicano@ua.pt (T.A.); joana.saraiva.vieira@ua.pt (J.V.); 2 Department of Physics and I3N, University of Aveiro, Campus Universitário de Santiago, 3810-193 Aveiro, Portugal; mfacao@ua.pt

**Keywords:** optical fibers, long period fiber grating, CO_2_ laser, coupled mode theory

## Abstract

Long period gratings (LPGs) inscribed in single mode fibers (SMFs) using CO_2_ laser irradiation were modelled numerically using the coupled mode method. The model considers the specifications of the inscription technique, such as the shape of the refractive index modulation that mimics the circularly symmetric point-to-point laser irradiation profile. A simple expression for predicting the resonant wavelength was obtained assuming a two-mode coupling model. However, to explain the spectra of the experimental LPGs, it was necessary to assume a reasonably high refractive index change and a multimode coupling model. Furthermore, using the developed model and a genetic algorithm to fit experimental resonances to simulated ones, we were able to estimate the maximum refractive index change, obtaining a value of 2.2 × 10^−3^, confirming the high refractive index change. The proposed model also predicts a second order resonance for this high value of refractive index change that was confirmed experimentally. Hence, with this model, we found some significant differences in the LPGs behavior when compared with conventional ones, namely, the emergence of coupling between different cladding modes and the competition of first and second order resonances which change the LPG transmission spectrum.

## 1. Introduction

Long period gratings (LPGs) inscribed in single mode fibers (SMF), with periods longer than 10 µm, promote the light coupling from the core guided mode to a specific cladding mode at the resonant wavelength [1], leading to attenuation dips in the transmission spectrum. These attenuation dips depend on the grating period and on the refractive index modulation. LPGs have many applications in both optical communications [2,3,4] and sensing [5,6,7,8] fields. In the sensing field, LPGs can be used as temperature, strain or refractive index sensors, furthermore, they can be made sensitive to chemical and biochemical parameters by coating with appropriate materials [9,10].

CO_2_ laser irradiation techniques have been used for fabrication of LPGs with good results. This technique does not require photosensitivity and can be used to fabricate LPGs in almost all types of fibers, including pure-silica core fibers and photonic crystal fibers (PCFs). Hence, compared to the UV irradiation technique, CO_2_ laser irradiation techniques are more flexible and have lower cost, maintaining the stability provided by the laser inscription techniques.

CO_2_ laser inscribed LPGs are typically written using the point-by-point method. Segments of the fiber are periodically exposed to 10.6 µm wavelength radiation from a CO_2_ laser, producing a localized fiber heating and a consequent refractive index change [11]. Several CO_2_ laser irradiation techniques to inscribe LPGs with antisymmetric and symmetric irradiation have been demonstrated and improved [2,11,12,13]. At the same time, studies on the physical mechanism involved in the refractive index change have been performed. The refractive index changes have been attributed to residual stress relaxation and/or glass densification [2,14,15], nevertheless, the process was not clearly explained yet. Furthermore, some methods were already used to measure the produced refractive index change but reported results are not in agreement with each other [15,16,17]. In fact, the refractive index change is an important parameter that defines LPG characteristics, which means accurate knowledge of this parameter is fundamental for LPGs modelling. Furthermore, LPG sensitivity to the surrounding medium is highly dependent on the coupled cladding mode [5,6,7]. By choosing the right coupling mode and the right period it is possible to design LPG-based sensors that are highly sensitive or even insensitive to a certain parameter [18]. In this way, LPGs modelling is fundamental for both sensor design and LPGs inscription planning.

Here, we report a detailed theoretical model, based on coupled mode theory, for CO_2_ laser symmetrically irradiated LPGs in SMFs that permit the estimation of the refractive index change from the LPG transmission spectrum. We use an automated LPG inscribing technique recently proposed in [13] that has the advantage of producing highly reproducible LPGs with a circularly symmetric refractive index change. The theoretical model takes into consideration the specifications of this inscription technique. The estimation of the refractive index change was done by fitting the simulation results to the wavelength resonances of the fabricated LPGs using a genetic algorithm (GA). 

## 2. CO_2_ Laser Irradiation Inscription Method

**Figure 1:**
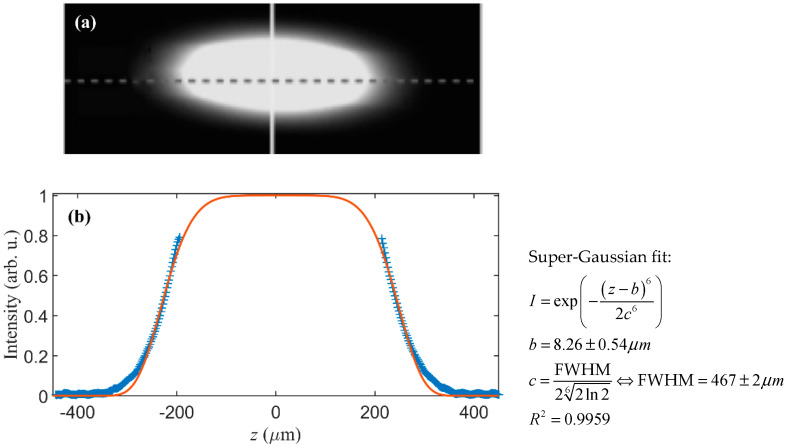
Beam heating profile in the fiber obtained with the LZM cameras (**a**) and correspondent super- Gaussian fit (**b**). The dots represent the collected data points and the red line represents a super-Gaussian fit.

The LPGs were inscribed using the technique proposed in [13], which uses a commercial CO_2_ laser fiber processing system (LZM-100 LAZERMaster from AFL Fujikura, Chessington, Surrey, UK). This point-by-point technique allows for constant rotation of the fiber during irradiation, leading to a uniform exposure around the fiber that results in a circularly symmetric refractive index change. This system produces LPGs with highly reproducible characteristics; however, the period must be higher than 800 µm to avoid overlapping the irradiated areas. The laser induced heating profile—which is produced in the fiber by the LZM system—is displayed in Figure 1. This profile was approximated by a super-Gaussian function of order *N* = 6 and a full width at half maximum (FWHM) of 467 µm. 

**Figure 2:**
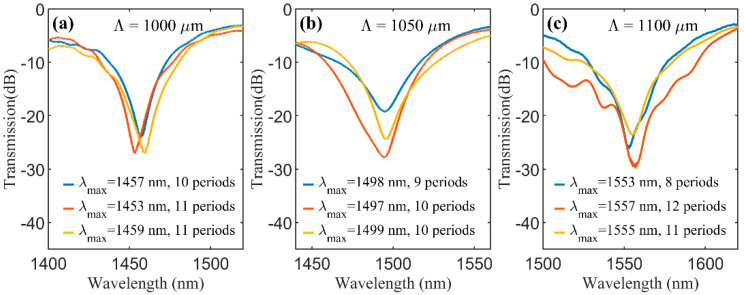
Transmission spectra of the produced long period gratings (LPGs) with periods (**a**) 1000, (**b**) 1050 and (**c**) 1100 μm.

We have inscribed LPGs in step index SMFs with different periods (1000, 1050 and 1100 μm). The point-by-point technique allowed us to monitor the LPG spectrum after each irradiation pulse, in such a way that the optimum length of the LPG was easily determined, i.e., the length that provides the maximum attenuation dip, which corresponds to the maximum coupling. Hence, the LPGs were produced with an optimized LPG length. For each period, three LPGs were produced to confirm the reproducibility of this technique. The LPGs transmission spectra were measured with an optical spectrum analyzer (OSA) (Q8384 from Advantest, Tokyo, Japan) in combination with a broadband source (WL-SC400-2, Fianium, Southampton, UK). The spectra of the LPGs produced are displayed in Figure 2. 

All spectra have attenuation dips with heights higher than 20 dB. The dips are similar in each set, having a maximum difference of 6 nm (Λ = 1000 µm) for the resonant wavelength and a maximum difference of 5.95 dB (Λ = 1100 µm) for the height of the dip. Furthermore, LPGs with larger period present higher resonant wavelengths.

## 3. Theoretical Model

In the case of a SMF, the refractive index perturbation of the LPG promotes the coupling between the fundamental core mode (HE_11_ or LP_01_) and the cladding modes of the fiber. The coupling interaction between these forward propagating modes can be described by the coupled mode theory. The general coupled-mode equations that describe the evolution of the mode amplitudes along the LPG (*z*) are written as [1,19]:

(1) $$\frac{dA_{p}}{dz}=i\sum_{p}A_{q}K_{qp}\exp(i\Delta \beta_{qp}z),$$

where *A_p_* and *A_q_* are the amplitudes of the mode field *p* and *q*, respectively. Δ*β_qp_* is the difference of the propagation constants of the modes, *z* is the propagation direction and *K_qp_* is the coupling coefficient between the modes *q* and *p*.

We assumed that the induced refractive index change has the same form as the fiber heating profile, i.e., the form of a super-Gaussian of degree *N* as displayed in Figure 1, and given by:

(2) $$\delta n(z)=\bar{\delta n}\;Y(z)=\bar{\delta n}\;\sum_{j}y\left(z-b_{j}\right),$$

with $\bar{\delta n}$ being the maximum value of the refractive index change, *b_j_* the position of the super-Gaussian peaks separated by the LPG period, Λ, and:

(3) $$y(z-b_{j})=\exp\left(-\frac{{\left(z-b_{j}\right)}^{N}}{2c^{N}}\right)\;\mathrm{with}\;c=\frac{\mathrm{FWHM}}{2\sqrt[{N}]{2\ln2}}.$$

The coupling coefficients can be defined as:

(4) $$K_{qp}=\sigma_{qp}Y(z).$$

Since the CO_2_ laser irradiation can induce refractive index changes in both the cladding and the core, *σ_qp_* is given by:

(5) $$\sigma_{qp}=\frac{\omega \varepsilon_{0}n_{co}\bar{\delta n_{co}}}{2}\iint_{\mathrm{core}}E_{q}E_{p}^{*}dxdy+\frac{\omega \varepsilon_{0}n_{cl}\bar{\delta n_{cl}}}{2}\iint_{\mathrm{cladding}}E_{q}E_{p}^{*}dxdy,$$

where $\varepsilon_{0}$ is the vacuum permittivity, *ω* the angular frequency, $n_{co}$ and $n_{cl}$ are the refractive indexes of the core and cladding, respectively, $\bar{\delta n_{co}}$ and $\bar{\delta n_{cl}}$ are their maximum changes and **E***_q_* and **E***_p_* are the electric field of the modes *q* and *p*. 

Considering the coupling between two modes, the coupled mode equations become:

(6) $$\begin{array}{l} \frac{dA_{1}}{dz}=i\sigma_{11}Y(z)A_{1}+i\sigma_{21}Y(z)A_{2}\exp\left(-i\Delta \beta z\right)\\ \frac{dA_{2}}{dz}=i\sigma_{22}Y(z)A_{2}+i\sigma_{12}Y(z)A_{1}\exp\left(i\Delta \beta z\right) \end{array}$$

where $\Delta \beta =\Delta \beta_{12}=\beta_{1}-\beta_{2}=-\Delta \beta_{21}$. The super-Gaussian series can be defined by a Fourier series:

(7) $$Y(z)=\sum_{n=-\infty }^{\infty }\alpha_{n}\exp\left(i\frac{2\pi n}{\Lambda }z\right)dz,$$

where the coefficients $\alpha_{n}$ are given by:

(8) $$\alpha_{n}=\frac{1}{\Lambda }\int_{-\frac{\Lambda }{2}}^{\frac{\Lambda }{2}}y(z)\exp\left(-i\frac{2\pi n}{\Lambda }z\right)dz.$$

Considering only the terms of order −1, 0 and 1 of the Fourier series (7), and neglecting the terms on the right-hand side of the differential Equations (6) that have rapid oscillations in *z* (synchronous approximation), yields:

(9) $$\begin{array}{l} \frac{dA_{1}}{dz}=i\sigma_{11}\alpha_{0}A_{1}+i\sigma_{21}\alpha_{1}\exp\left(i\frac{2\pi }{\Lambda }z-i\Delta \beta z\right)A_{2}\\ \frac{dA_{2}}{dz}=i\sigma_{22}\alpha_{0}A_{2}+i\sigma_{12}\alpha_{-1}\exp\left(-i\frac{2\pi }{\Lambda }z+i\Delta \beta z\right)A_{1} \end{array}$$

Since $\sigma_{kj}=\sigma_{jk}^{*}$, $\alpha_{1}=\alpha_{-1}$, and assuming the initial conditions $A_{1}(0)=1$ and $A_{2}\left(0\right)=0$, the solution for the above system, written in terms of power, is

(10) $$\begin{array}{c} P_{1}={\left|A_{1}\right|}^{2}=\cos^{2}\left(\gamma_{0}z\right)+{\left(\frac{\delta }{\gamma_{0}}\sin\left(\gamma_{0}z\right)\right)}^{2}\\ P_{2}={\left|A_{2}\right|}^{2}={\left(\alpha_{1}\frac{\left|\sigma_{12}\right|}{\gamma_{0}}\sin\left(\gamma_{0}z\right)\right)}^{2}\\ \mathrm{with}\;\delta =\alpha_{0}\left(\sigma_{11}-\sigma_{22}\right)/2+\Delta \beta /2-\pi /\Lambda \;\mathrm{and}\;\gamma_{0}=\sqrt{\alpha_{1}^{2}{\left|\sigma_{12}\right|}_{}^{2}+\delta^{2}} \end{array}$$

With these solutions, we may estimate the resonant wavelength given by δ=0, i.e.,

(11) $$\alpha_{0}\frac{\left(\sigma_{11}-\sigma_{22}\right)}{2}+\frac{\pi \Delta n_{eff}}{\lambda_{\max,1}}-\frac{\pi }{\Lambda }=0$$

where $\Delta n_{eff}=n_{eff,1}-n_{eff,2}$.

The above solutions correspond to first order coupling. However, our experimental results and integration of (6) with *Y*(*z*) up to first order showed a reasonable coupling for a shorter wavelength, which we attributed to second order coupling, given by:

(12) $$\alpha_{0}\frac{\left(\sigma_{11}-\sigma_{22}\right)}{2}+\frac{\pi \Delta n_{eff}}{\lambda_{\max,2}}-\frac{2\pi }{\Lambda }=0$$

This coupling cannot be explained within the synchronous approximation and numerical tests which revealed that for large *δn* it happens for reasonable LPG lengths, but for small *δn*, it occurs for very large lengths which are experimentally unfeasible. Similar higher diffraction order has been reported in previous works [20].

## 4. Results

We calculated the mode power evolution in the LPG for each wavelength and its full spectrum through full integration of system (1) considering one and several cladding modes, as indicated in each section, and the full super-Gaussians given in (2). To predict the relation between grating period and resonant wavelength we have used the expressions (11) and (12). We assumed a bare standard SMF on air composed by a cladding of pure silica with a radius of 62.5 µm and a core of GeO_2_ doped silica with a concentration of 3% and a radius of *r* = 4.1 µm. The cladding and core refractive indexes were calculated using the Sellmeier coefficients as in [21], which are 1.4440 and 1.4485 at 1550 nm.

The electric field distribution (E) and the effective refractive indexes (*n_eff_*) were obtained using the software package Comsol Multiphysics^®^ with the Wave Optics module that solves the full vectorial Helmholtz equation. We calculated the fundamental core mode (LP_0,1_) and 200 cladding modes, the ones with the highest effective refractive indexes. Among those modes, we selected ten modes with the same azimuthal symmetry of the core modes (HE_1,m_), the correspondent effective refractive indexes at 1550 nm are in Table 1. Note that in gratings with symmetric index changes like the ones that are being studied here, the coupling may only occur between modes with the same azimuthal symmetry [1]. 

We made preliminary numerical tests considering a uniform refractive index change in the core or its symmetrical in the cladding. These tests yielded similar results in both cases. During all our numerical calculations, we have opted to consider only a uniform refractive index change in the cladding since, this should be closer to the actual change induced by our inscription method. 

The system of differential Equations (1) was solved using a Runge–Kutta method. In order to calculate the spectral response of the LPGs, we repeated the integration for the same period but for several wavelengths, and for each wavelength the power at *z* = *L*_max_ was considered, where *L*_max_ is the length at which the power of the fundamental mode attains its minimum at the resonant wavelength. 

### 4.1. Coupling between Cladding Modes

**Figure 3:**
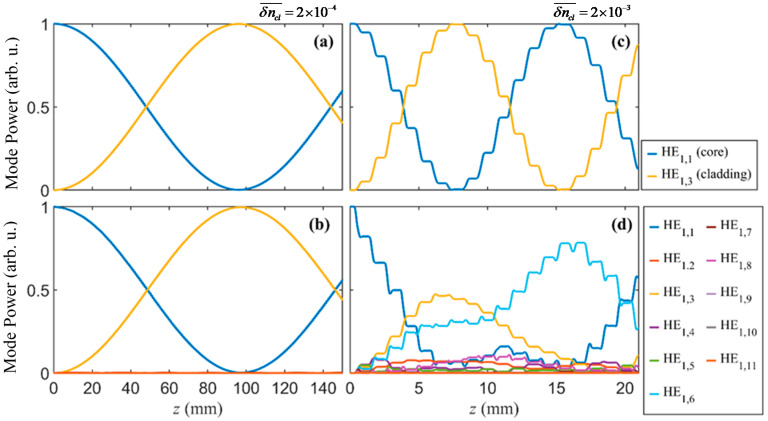
Power evolution in an LPG calculated with one (**a**,**c**) and ten (**b**,**d**) cladding modes simultaneously. A $\bar{\delta n_{cl}}=2\times 10^{-4}$ with a period of 821 μm (**a**,**b**) and a $\bar{\delta n_{cl}}=2\times 10^{-3}$ with a period of 1117 μm (**c**,**d**) and a wavelength of 1550 nm were considered.

In [1,22] it was demonstrated that coupling between the different cladding modes is usually negligible, such that the transmission spectrum can be calculated by overlapping all the individually calculated spectra for each cladding mode. However, in those works the gratings were weak, i.e., the refractive index change was less than 1 × 10^−4^. In the case of CO_2_ laser inscribed LPGs, the refractive index change can reach higher values, with a value of ~10^−3^ already having been reported [16]. These high values increase the coupling coefficients to the point that the coupling between different cladding modes is not negligible. An example of this different behavior for small and large refractive index modulation is represented in Figure 3. There, we present the mode power evolution at 1550 nm for $\bar{\delta n_{cl}}=2\times 10^{-4}$ (Figure 3a,b) and $\bar{\delta n_{cl}}=2\times 10^{-3}$ (Figure 3c,d), for LPGs whose period is given by (11) when considering HE_1,3_. The results in Figure 3a,c, were obtained by considering only one cladding mode, whereas the results in Figure 3b,d were obtained by including multiple cladding modes, namely, the ten first azimuthally symmetrical cladding modes. Figure 3a,b are identical, however, there is a clear difference between graphs in (c) and (d), showing coupling between the different cladding modes and the appearance of a second order resonance for the same period and same wavelength (associated with mode HE_1,6_).

**Figure 4:**
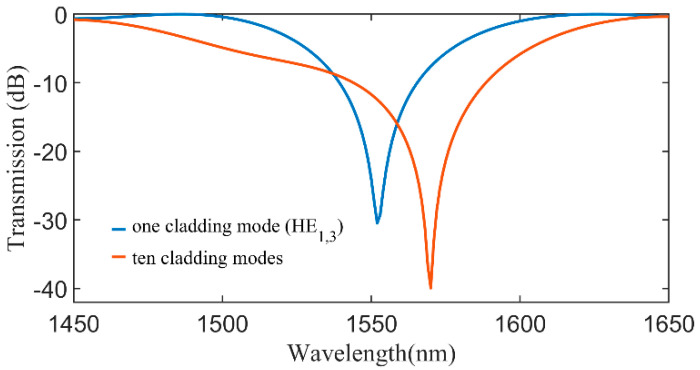
Transmission spectra calculated considering only two modes or the ten first azimuthally symmetrical cladding modes, a $\bar{\delta n_{cl}}=2\times 10^{-3}$, a period of 1117 µm and length of 7 periods.

To verify the differences in the transmission spectra, we also determined them for $\bar{\delta n_{cl}}=2\times 10^{-3}$ in both situations (one and ten cladding modes). The results are displayed in Figure 4 and show that the resonant wavelength changes with the number of modes are considered in the simulation. Note that, in both cases, we have used LPG optimal lengths (7.8 mm which corresponds to 7 periods) which is the number of periods closer to the length at which core mode power reaches a minimum on the graphs in Figure 3. These results reveal that Equation (11) is not very accurate for determining the resonant wavelength in cases of large refractive index changes, since we cannot consider only coupling between two modes but instead, we need to consider several modes to accurately model the LPGs. Nevertheless, it is good as a first approximation, as we show in sub Section 4.3. There are thousands of modes supported by the cladding, thus it is not possible to consider all of them in the simulations. However, in Figure 3d we can see that modes higher than the HE_1,7_ do not couple significantly. In this way, hereafter we are considering the same 10 cladding modes simultaneously in all simulations.

Another aspect of the power evolution that is observable in the graphs of Figure 3c,d is the step-like curves, which are not visible for lower refractive index changes. Each step corresponds to a period, the steady part corresponds to the region not irradiated, with zero refractive index change, and the part where the mode power changes corresponds to the irradiated region where the refractive index is changed. 

### 4.2. Refractive Index Change Estimation

As mentioned above, accurate knowledge of the refractive index change enables the modelling of LPGs characteristics which is important to guide the fabrication process. 

Here, we estimate the maximum refractive index change in the cladding, considering uniform refractive index change, using a genetic algorithm (GA) that fits the resonances of the fabricated LPGs to the numerically determined resonances using the model presented in Section 3. 

**Figure 5:**
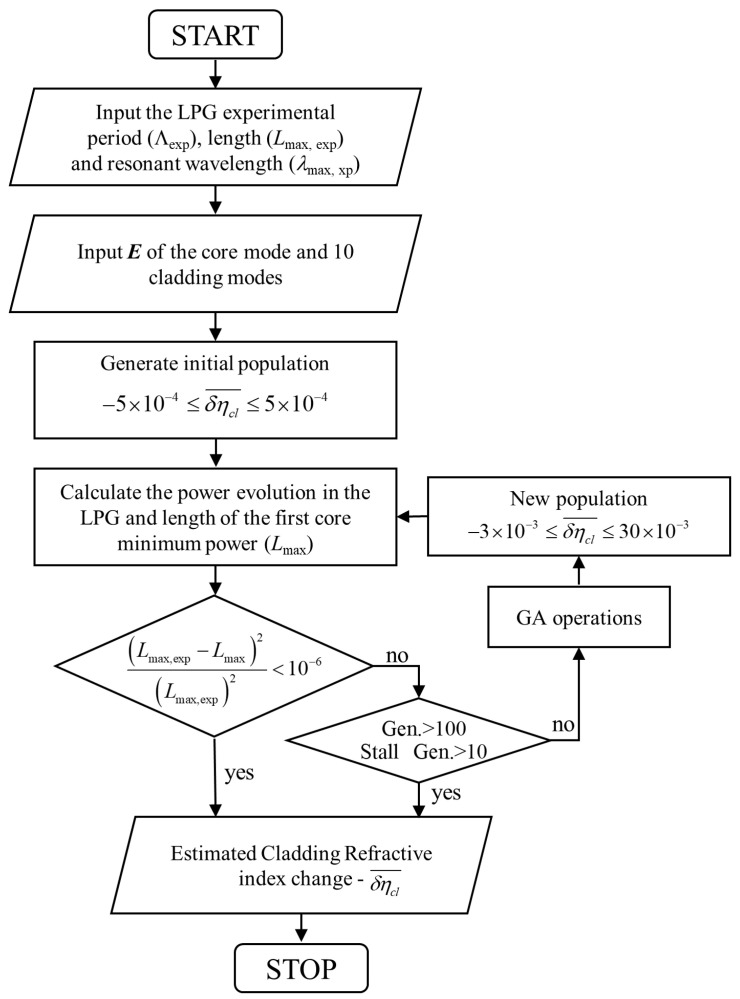
Flow chart of the GA implemented for the refractive index change estimation.

The GA was implemented in order to find the refractive index change for which the numerical optimal LPG length better approximates the experimental optimal LPG length in the resonant wavelength. For each LPG period, we have considered the averages of the resonant wavelengths and of the lengths of the three produced LPGs (Figure 2). The algorithm flow chart is shown in Figure 5 where we also show some of the GA conditions that have been used and were chosen after some preliminary tests. Within the GA routine, we solve the differential equations system (1) for the core mode and the first ten azimuthally symmetrical cladding modes and the full series of super-Gaussians given in (2), considering the experimental LPG periods and their resonant wavelengths. We then find the LPG length that corresponds to the first minimum of the core mode power and compare it with the experimental LPG length. We used a GA routine from Matlab and since the GA uses initial populations and selection methods that rely on random distributions, each run may produce slightly different results. For that reason, we ran it three times and the results are presented in Table 2. We obtained similar refractive index changes for all three sets of experimental LPGs, and in all cases the coupling was mainly with the mode HE_1,3_. Calculating the average of all the refractive index changes obtained with GA (see Table 2), we estimate a value of 2.2 × 10^−3^. Although this value is high, it is within the range of the reported values [16].

### 4.3. First and Second Order Wavelength Resonances and Experimental Validation

**Figure 6:**
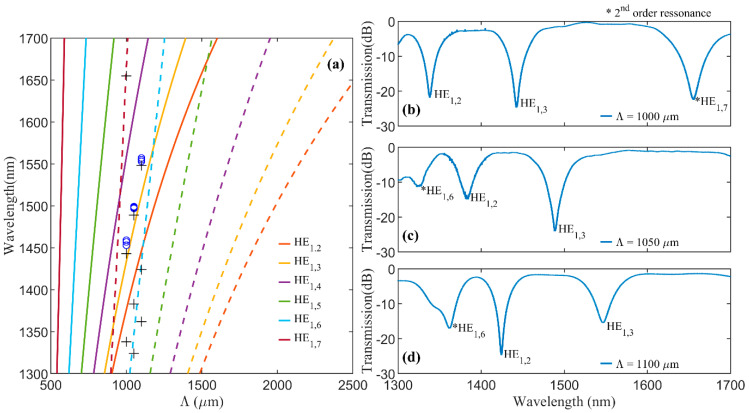
Resonant wavelengths as a function of the grating period (**a**) for the first (solid lines) and second order (dash lines) coupling of azimuthally symmetrical cladding modes HE_1,2_ to HE_1,7_. Markers represent the experimental resonant wavelengths of the produced LPGs that (o) correspond to the attenuation dips of the spectra of Figure 2 and (+) to the attenuation dips of the transmission spectra of LPGs produced with a periods of (**b**) 1000, (**c**) 1050 and (**d**) 1100 µm and a length of 14, 14 and 19 periods, respectively. The lengths of the LPGs were chosen in order to show more dips.

Using the estimated refractive index change and the expressions (11) and (12), we were able to estimate a relation between the LPG period and the first and second orders’ resonant wavelengths for a series of azimuthally symmetrical cladding modes (Figure 6a). As expected, the resonant wavelengths of the fabricated LPGs (Figure 2) are close to the HE_1,3_ mode line. We verified that, considering the first order coupling, longer periods promote the coupling with lower-order modes while shorter periods promote the coupling to higher-order modes. Furthermore, Figure 6a indicates that, assuming coupling with the same cladding mode, the resonant wavelength increases with increasing periods. The second order presents the same behavior but for much longer periods. In the points where the curves of the first order and second order resonances cross each order, coupling with the two modes that cross should occur with one mode in first order and the order one in second order, as shown in Figure 3d. However, the maximum coupling of each mode/order may occur for different LPG lengths.

To validate the numerical analysis, we fabricated another set of LPGs and measured their transmission spectra for a wavelength range from 1300–1600 nm (Figure 6b). The LPGs were fabricated with periods of 1000, 1050 and 1100 µm, as well as lengths chosen in order to show more dips. Besides the attenuation dips that correspond to the ones in Figure 2, the spectra present additional dips. By comparing the transmission spectra of these LPGs with the expected resonant wavelengths of Figure 6a, we identified the cladding modes that should be associated with each dip, verifying that some dips should correspond to first order and others to second order resonances. 

The small difference between the experimental resonant wavelengths and the calculate ones is partly due to the use of (11) and (12), as explained in sub Section 4.1. Nevertheless, these results show that our model together with the estimated refractive index change can provide a good first approximation of the LPGs behavior. 

## 5. Conclusions

The mode power transfer in LPGs inscribed in SMFs by the CO_2_ laser irradiation technique was studied using a theoretical model, based on the coupled mode method. Taking into consideration the specifications of the inscription technique, the model assumes azimuthally symmetric refractive index changes, in the form of a train of super-Gaussian profiles with high maxima that mimics the circularly symmetric point-to-point laser irradiation profile. We started by demonstrating that for high refractive index changes the coupling between different cladding modes is not negligible. To fully explain the spectra of the produced LPGs, several cladding modes needed to be considered simultaneously. Nevertheless, we have used a two-mode coupling model within the synchronous approximation to obtain a simple relation that approximates the dependency of grating periods and resonant wavelengths. Using a genetic algorithm, we then developed a method to estimate the refractive index change. By fitting the wavelength resonances of several produced LPGs to numerically obtained ones using a multi-mode coupling model, we were able to estimate the refractive index change maximum to approximately 2.2 × 10^−3^. Using the estimated refractive index change, we predicted the resonant wavelengths for several cladding modes in a range of LPG periods. The experimental results were in good agreement with the predicted resonances. Furthermore, we numerically demonstrated the existence of a second order resonance that, for the same grating period, occurs at shorter wavelengths. These second order resonances were confirmed by the experimental results.

## Figures and Tables

**Table 1:** Effective refractive indexes of the core mode and the chosen 10 cladding modes at 1550 nm.

Mode	*n_eff_*
HE_1,1_	1.4457
HE_1,2_	1.4439
HE_1,3_	1.4438
HE_1,4_	1.4435
HE_1,5_	1.4430
HE_1,6_	1.4425
HE_1,7_	1.4418
HE_1,8_	1.4410
HE_1,9_	1.4402
HE_1,10_	1.4392
HE_1,11_	1.4380

**Table 2:** Estimated refractive index change obtained with the genetic algorithm (GA).

LPGs Experimental Values			Estimated Refractive Index Change in the Cladding $\left(\bar{\delta n_{cl}}\right)$ (×10^−3^)
Λ (µm)	*λ*_max_ (nm)	*L* (mm)	
1000	1456	10.7	2.37
			2.35
			2.40
1050	1498	10.2	2.29
			2.25
			2.25
1100	1555	11.4	2.02
			2.05
			1.93
